# The Western Diet–Microbiome-Host Interaction and Its Role in Metabolic Disease

**DOI:** 10.3390/nu10030365

**Published:** 2018-03-17

**Authors:** Marit K. Zinöcker, Inge A. Lindseth

**Affiliations:** 1 Department of Nutrition, Bjørknes University College, Lovisenberggata 13, 0456 Oslo, Norway; 2 Balderklinikken, Munchsgate 7, 0165 Oslo, Norway; inge.lindseth@balderklinikken.no

**Keywords:** western diet, metabolic disease, ultra-processed food, microbiome, inflammation, additives, acellular nutrients, whole foods, food industry, dietary guidelines

## Abstract

The dietary pattern that characterizes the Western diet is strongly associated with obesity and related metabolic diseases, but biological mechanisms supporting these associations remain largely unknown. We argue that the Western diet promotes inflammation that arises from both structural and behavioral changes in the resident microbiome. The environment created in the gut by ultra-processed foods, a hallmark of the Western diet, is an evolutionarily unique selection ground for microbes that can promote diverse forms of inflammatory disease. Recognizing the importance of the microbiome in the development of diet-related disease has implications for future research, public dietary advice as well as food production practices. Research into food patterns suggests that whole foods are a common denominator of diets associated with a low level of diet-related disease. Hence, by studying how ultra-processing changes the properties of whole foods and how these foods affect the gut microbiome, more useful dietary guidelines can be made. Innovations in food production should be focusing on enabling health in the super-organism of man and microbe, and stronger regulation of potentially hazardous components of food products is warranted.

## 1. Ultra-Processed Foods as Drivers of Diet-Related Disease

The share of whole versus ultra-processed foods is a dietary factor that has traditionally been given minor attention in nutritional science. However, a growing body of epidemiological research supports the idea that ultra-processed foods are detrimental to human health. Classification of foods according to processing has, among others, been reviewed by Monteiro and co-workers in the NOVA classification, where foodstuffs are grouped into categories according to the extent and purpose of processing applied to them [1]. The most extensively processed foods, termed “ultra-processed”, have been defined as “industrial formulations made entirely or mostly from substances extracted from foods (e.g., oils, fats, sugar, starch, and proteins), derived from food constituents (e.g., hydrogenated fats and modified starch), or synthesized in laboratories from food substrates or other organic sources (e.g., flavor enhancers, colors and several food additives used to make the product hyper-palatable)” [2]. The utility of the NOVA classification has been subject to discussion, as this classification is not based on unequivocal, distinct physical/chemical aspects of foods [3]. However, the NOVA classification has been acknowledged in several reports from The United Nations (UN) and World Health Organisation agencies, as well as in scientific journals [4]. Also, it is safe to conclude that ultra-processed foods are one major hallmark of the Western diet. In high-income countries, ultra-processed foods are now dominating the food supply, and they are rapidly gaining ground in growing economies [5]. Transnational food and beverage corporations are increasingly targeting low income countries, strategically using pricing and availability to increase consumption of ultra-processed foods at the expense of traditional foods [6]. Individuals consuming large amounts of these types of foods are at greater risk of being obese than people who consume relatively little [7,8,9], and the availability of ultra-processed foods is positively associated with the prevalence of obesity [10,11]. Also, the dietary share of ultra-processed foods determines the nutritional quality of diets in several populations [12,13,14].

Associations with adverse health effects in humans are also present when looking at food groups. For instance, although a high intake of meat in general is associated with an increased risk for several health outcomes [15,16,17,18,19], processed meat shows a much stronger association [20,21,22,23]. Intake of whole grains is associated with a reduced risk of several non-communicable diseases, as opposed to refined grains [24,25]_._ A study on fish intake reported adverse effects on markers of metabolic syndrome from processed fish, whereas whole fish seems to be protective [26]. These studies indicate that by studying how processed foods and whole foods affect human physiology and metabolism differently, we can shed light on the mechanisms of the adverse effects of the Western diet. Nutrition researchers have tended to focus their attention on characteristics of the Western diet such as the energy density and the addition of fat, sugar and salt. We argue that other factors introduced during food processing can have an equally important effect by promoting inflammation-related processes through diet–microbiome–host interactions.

## 2. Factors that Promote Inflammation through Diet-Microbiome-Host Interactions

The microbiota composition can change rapidly upon dietary changes [27] and contribute to negative health effects, as evidenced by microbe transplant studies in rodents. Such changes can include dysbiosis and microbiota encroachment, leading to inflammation and metabolic disturbances [28,29,30]. However, a change in microbiota composition is not a prerequisite for changes in function. Dietary factors that alter the metabolic behavior of the microbes already present can also have a large impact. Gut bacteria adjust their metabolism according to both the substances produced by other microbes and the nutrient supply, which may produce effects that influence metabolic and inflammatory pathways in the human body [31]. For instance, both human and animal studies have demonstrated that pathogens, pathobionts and other members of the microbiome can respond to a change in their environment (e.g., the presence of some emulsifiers; discussed later) by increasing their expression of virulence factors [32,33,34], thereby increasing the pro-inflammatory potential of the microbiome.

Major changes in the diet and subsequent changes in microbiota can, at least to some extent, be reversed within the same generation. However, recent studies in rodents have demonstrated that the loss of microbiota diversity due to dietary changes can be transferred to later generations, with progressive loss of diversity [35]. Also, a Western diet could lead to a permanent loss of bacteria important to microbiome function [36] and possibly induce inheritable metabolic changes via the epigenome [37]. In sum, the environment created in the gut by ultra-processed foods could be an evolutionarily unique selection ground for microbes with behaviors that promote diverse forms of inflammation-related disease.

## 3. Acellular Nutrients—A Major Shift in Our Diets

Throughout the human evolution, nutrients had to be released from cells (with a few exceptions, such as nutrients in milk, honey and eggs) to be available for uptake by the enterocytes. However, in a Western diet, a large share of the energy is provided by acellular nutrients*;* a term coined by Ian Spreadbury [38]. Acellular nutrients, i.e., nutrients not contained in cells, provide microbial and human cells with more easily digestible substrates [39] that influence human absorption kinetics [40,41,42] and are likely to influence intestinal bacterial growth [38].

The share of cellular nutrients that become accessible during digestion in the gut differs from food to food, depending on the nature of the food matrix and processing that alters the properties of the outer cell [39]. Animal cells lack cell walls, and their phospholipid membranes are efficiently hydrolyzed by human digestive enzymes, liberating the cell content into the gut lumen [43]. Processing of animal foods (e.g., mincing of meat) can break the cell structures and increase the total availability of nutrients [44].

Plant cells have cell walls consisting mainly of fibre [45], making the contents of intact plant cells less available for uptake in the small intestine. During mastication, some plant cells are ruptured, and the macromolecules inside become available for hydrolyzation by human digestive enzymes in the gut. The release of nutrients from ruptured cells is dispersed along a large part of the small intestine, and, depending on the particle size and the food matrix, a fraction of the cellular nutrients will not be released at all, passing through the small intestine [41,42]. Nutrients in intact plant cells reaching the colon will become available to fibre-degrading bacteria that hydrolyze the cell walls of the intact cells.

Food processing that leads to breakage of cell walls affects the nutrient availability in the small intestine. Where whole grains of, for instance, wheat contain all of their nutrients in cells, grains that are milled to whole meal flour will contain a mixture of intact and ruptured cells, as milling of plant seeds causes a substantial portion of the cells to rupture [39]. Further refining of the seed contents will leave less of the cells intact, and, in white flour, a large share of the nutrients is acellular. Although milling was also used by pre-agricultural humans, the level of consumption of refined acellular macronutrients from milled grains during the last centuries, and especially the last five decades, is by far unprecedented in a historical context. The production of modern, ultra-processed foods also highly relies on extracted oils and starches for use as ingredients in food products. Extraction of oils and starches from seed crops renders all the nutrients acellular. The possible impact of this increased accessibility has received little attention as of today, but the unarguable large shift in the nutrient accessibility cannot be discounted in terms of possible health impacts in light of the new knowledge of the microbiome.

Increased amounts of readily accessible acellular nutrients in a Western diet might facilitate increased growth potential as well as altered composition and metabolism of the gut microbiota. It is likely that an abundance of accessible nutrients in the small intestine of animals signals a large nutrient availability and sufficient resources to expand the bacteria’s territory [32,46]. These effects could propagate further down the digestive system. Accordingly, Turnbaugh and co-workers found that a Western diet high in simple sugars caused a dramatic loss of microbial diversity in mice, coinciding with a bloom in bacteria capable of metabolizing such sugars, which are normally not present in the distal colon [30].

Territory expansion signaling could translate to increased production of virulence factors, which could damage the host directly or via increased amounts of microbes or microbial products entering the bloodstream or the intestinal wall. Indeed, increased amounts of microbes and microbial by-products, originating from the gut, are found in patients with lifestyle diseases [47,48]. Also, the duodenal microbiota differs in composition and function in normal weight as compared to obese people, providing an indication that the small intestinal microbiota is a discrete determinant of body fat mass amount, and that manipulating the duodenal microbiota, for example through diet changes, could affect fat mass [49].

Further, research has shown that, for instance, starch from grain based foods [40] and fructose from sugary drinks [50] can exceed the carbohydrate absorption capacity of the small intestine in humans and mice, respectively, providing easily accessible growth substrates for the bacteria in the distal small intestine as well as in the colon.

Cellular plant foods are likely to affect the gut microbiota in a different way. As a substantial amount of cells from whole plant foods will enter the colon in an intact state [41], this could favor the growth of bacteria that degrade fibre and produce beneficial metabolites (e.g., short chain fatty acids (SCFA)) in the competition for substrates in the human gut.

We argue that the increase in small intestinal nutrient availability from acellular, compared to cellular, foods could prove to be one decisive factor for the microbiota-mediated effects of the diet, and that the protective effects from whole plant foods on several health outcomes could be partly explained by favoring the growth of beneficial fibre-degrading bacteria in the colon.

The number of studies that have directly addressed how acellular versus cellular foods affect physiology is limited. To advance this field further there is a need for detailed investigation into how acellular and cellular meals and diets affect the microbiome in the gastrointestinal tract.

## 4. Food Additives

The number of food additives approved for use by the industry has been soaring over the last few decades [51]. The risk assessment conducted prior to the approval of such substances does generally not include effects on the microbiome [51], but recent studies have indicated that additives can induce microbiota-mediated adverse effects in the host (Table 1).

The increasing use of emulsifiers in food production, e.g., to improve foods’ sensory properties in low-fat formulations [52], is partly driven by innovation in specialty products for health-conscious consumers [53]. However, several studies have reported altered microbiota composition and gut inflammation in rodents fed commonly used emulsifiers [29,54,55,56].

Recent publications report that emulsifiers can act by increasing virulence factors and thereby the pro-inflammatory potential of the microbiome [33,34], and that this low grade inflammation caused by emulsifiers can promote colon carcinogenesis [57]. The accumulating data linking selected emulsifiers to gut inflammation should cause concern and call for precautionary action. However, lecithin, a substance of animal and plant tissue, which also acts as an emulsifier, has been suggested as a therapeutic agent in treatment of inflammatory bowel disease, as it could improve the re-establishment of an intact mucosal barrier in humans [58]. The contradictory effects of different types of emulsifiers underline the importance of thorough testing of every food additive on the gut microbiota before approval.

Non-caloric artificial sweeteners (NAS) have made their way from foul-tasting soft drink alternatives for diabetics, to become the choice of the masses. These sweeteners have also not been tested for effects on the gut microbiota prior to approval. Effects from rodent studies include altered composition of microbes leading to impaired glucose tolerance [28,59,60] as well as increased pro-inflammatory potential [61,62] and liver inflammation [61]. NAS-induced alterations in the microbiota composition leading to adverse metabolic outcomes should give rise to concern, especially because the demand for NAS-containing products are increasing, and persons suffering from metabolic disorders could be more likely to choose such products for health benefits. Rodent studies demonstrating the disruptive properties of artificial sweeteners on gut microbiota have been criticized for using concentrations corresponding to the acceptable daily intake (ADI) in humans, thereby exceeding a normal consumption pattern. However, the ADI is set to include a safety margin, usually by dividing the “no observed adverse effect level” by a factor of 100. An observed effect at the ADI level indicates an insufficient safety margin and calls for reassessment.

## 5. Other Components of the Western Diet that Influence Inflammation

A Western diet can lead to increased levels of endotoxin-producing bacteria in the intestinal tracts of both humans and mice, resulting in metabolic endotoxemia [65,66]. Several studies investigating the effect of major dietary changes on microbiota in mice have utilized so-called high fat diets, and negative changes—including metabolic endotoxemia—are often attributed to the fat content [67]. However, several other dietary factors could be contributing. An animal study demonstrated that changes in the microbiota leading to intestinal inflammation were caused by a lack of fermentable fibre, and not by the content of fat in the diet [68]. When mice were fed a diet that induced metabolic endotoxemia, adding a prebiotic improved metabolic marker [69]. Correspondingly, dietary polyphenols have shown the ability to restore gut barrier integrity [66], attenuate a number of inflammatory effects of a so-called high fat diet and to induce a healthy microbiota profile in mice when fed together with the high fat diet [70]. Thus, the Western diet’s contribution to inflammation in experimental settings could partly be explained by the low content of plant-derived nutrients, like fibre and phytochemicals.

Furthermore, diet-induced inflammation could be mediated partly by the PAMPs (pathogen associated molecular patterns) produced by microbes in processed foods [71]. PAMPs, (e.g., lipopolysaccharide (LPS) and other toll like receptor stimulants) arise from bacterial growth during periods between food preparation and heat treatment, which are likely to be prolonged in industrial processing compared to home cooking. When fresh whole foods are prepared for processing, such as mincing meat or dicing vegetables, the increase in surface area and access points favors the conditions for the growth of bacteria. Clett Erridge has demonstrated that both minimally processed (e.g., diced onions and minced meats) and more processed foods (e.g., cheeses, ice cream and chocolate) contain PAMPs capable of inducing the release of pro-inflammatory cytokines in vitro [72,73]. As these PAMPs are heat stable, they can be present after heat treatment in food products with seemingly good microbiological quality [72]. A study of human volunteers reported a reduction in white blood cell count, low density lipoprotein cholesterol, body weight and waist circumference after one week on a diet low in PAMPs, whereas the beneficial changes were reversed by a diet high in PAMPs [71]. If these effects are corroborated by more studies, then the quality assessment of industrial food products should include analyses for LPS and other toll-like receptor stimulants to avoid inflammatory responses in the host, and improved measures should be taken to limit growth of PAMP-producing bacteria during processing. As the microbiological quality of foods is generally assessed by the presence of live bacteria, the presence of inflammation-inducing bacterial molecules could be a potential food safety issue that has gone under the radar.

Reversely, probiotic or fermented foods could contain beneficial bacterial molecules left in the foods during fermentation. Studies have shown beneficial effects of fermented milk products on metabolic markers in mice [74,75,76], also independently of the presence of live probiotic bacteria, either in the product or in the gut of the recipient [77]. It has been demonstrated in vitro that metabolites from probiotic bacteria are capable of reducing the release of pro-inflammatory cytokines [78,79,80]. The exact molecules involved are yet to be identified. We suggest that bacteria-derived molecules in foods are important signals, both to the microbial community in the gut and the immune system of the host, capable of altering microbiota behavior and modulating host inflammatory responses. Hence, types of processing that generate beneficial or detrimental bacterial molecules in foods could be a relevant addition to the discussion regarding food processing.

Many foods undergo extensive heat treatment during preparation that leads to the generation of advanced glycation end products (AGEs). AGEs increase with an increased cooking time, temperature and the absence of moisture [81]. Processed foods high in fat and protein typically contain higher levels of AGEs than carbohydrate-rich foods; however, the processing of breakfast cereals and carbohydrate-rich snacks that involve extrusion and high temperatures also leaves high amounts of AGEs in the finished product [81]. The majority of dietary AGEs escape absorption in the small intestine and become substrates for microbes, capable of modifying the composition of the host microbiome both in rodents and in humans, as measured in feces [82]. Dietary restriction of AGEs can alter the microbiota composition in humans [83], but, at present, we are not aware of interventions that have investigated whether AGE-induced changes in the microbiome lead to changes in markers of disease in humans. In rats, a diet high in AGEs led to reduced levels of strains associated with positive health effects, increased levels of species associated with detrimental health effects [84,85] as well as increased colon permeability [85], but there are rodent and in vitro studies where the results appear contradictory to this [84,86,87,88]. Due to the heterogeneity and the multitude of AGEs that arise from the heat treatment of processed foods and their apparent contradictory effect, heat-treatment procedures that could have a potential detrimental effect on the gut microbiota warrant further investigation.

## 6. Diet-Induced Inflammation and Its Role in Metabolic Disease

To what degree findings from animal studies, which largely form the basis of this paper, are relevant for the development of metabolic disease in humans is an important consideration, and data from human studies are still scarce. However, samples from the appendix of morbidly obese patients showed adverse changes in microbiota composition compared to healthy persons [89]. The connection between gut dysbiosis, inflammation and metabolic disease is also strengthened by human studies showing gut dysbiosis in prediabetic [90], diabetic [91,92] and obese patients [93,94].

Diet-induced inflammation-related processes could interfere with our biologically fine-tuned systems for nutrient sensing and energy balance and cause food cravings and hyperphagia. In mice, withdrawal from a Western style diet caused anxiety as well as cravings of sweet, energy dense foods [95]. Also, chronic exposure to LPS in rats reduces the effect of the satiety-inducing gut peptide cholecystokinin, as well as the signaling effect of leptin on the vagus nerve [96,97]. These data are shedding some eagerly awaited light on the mechanisms of leptin resistance and can provide an explanation for the imbalance in energy intake and energy expenditure coinciding with gut inflammation as observed in mice [29]. Accordingly, a recent study reported that diet-induced dysbiosis increased gut inflammation and altered vagal gut–brain communication in rats, along with an increase in body fat accumulation [98]. Reversely, a gut microbiota fed a healthy diet produces substances that influence satiety in a positive manner. In humans, it has been shown that SCFA from the fermentation of dietary fiber activate receptors throughout the small intestine and the colon, leading to increased release of satiety-inducing gut peptides [99], and several human studies have demonstrated increased satiety and reduced hunger after the addition of fermentable carbohydrates to the diet [100]. Hence, a Western diet that induces gut-derived inflammation in the host could disturb mechanisms for maintaining energy balance and contribute to obesity and subsequent metabolic disease.

## 7. Why Nutrition Experts Disagree on Dietary Advice

The fact that the majority of the evidence underlying nutrition policies and dietary advice in most countries has been interpreted in a context not involving the microbiome can explain some of the persistent conflicting issues in nutritional science, for instance, regarding the role of dietary fats in health. Recent studies have demonstrated that gut bacteria have a significant influence on lipid metabolism in mice, and that hypercholesterolemia and atherosclerosis are affected by functional changes in gut microbiota [101,102]. Also, the lack of suitable diets in animal studies is a further complicating matter. A study evaluating the use of experimental diets in 35 animal studies found that only 14 per cent were using appropriate diet comparisons [103]. We argue that there is a need to re-investigate the effects of dietary fats on human health parameters, applying fatty acid compositions actually present both in ultra-processed and whole foods and interpreting them in a microbial context.

The microbial angle could also offer a plausible explanation for the conflicting outcomes of human studies of health effects from fibre and whole grains [104,105]. In whole grains, the fibre is part of the cellular structures of the food, whereas “fibre” is very broadly defined, and can, in dietary studies, refer to heterogeous food components, e.g., intact fibre in whole plant foods, bran as a byproduct of milling or fibres formed during extensive processing like extrusion [106]. Extrusion of cereal foods utilizes high heat and high pressure, and the process leads to several chemical and physical changes, including inactivation of endogenous enzymes, increased content of soluble dietary fibre and mechanical damage to the cell walls [107]. Fibre-rich foods originating from extruded grains might act very differently from fibre-rich whole foods, as demonstrated in pigs. When pigs were fed extruded grains, their microbiota changed towards a less diverse and less beneficial composition compared with the pigs that were fed untreated grains [107].

## 8. A Complex Problem with a Simple Solution

We argue that while we are striving to make sense of the diet–microbiome–host interaction and its role in human health, we already know enough to provide better dietary advice on how to better nourish our super-organism.

A large body of research now supports the hypothesis that the Western Diet is causing changes in gut microbiota associated with obesity and metabolic disease [67]. There also seems to be consensus that fixing the diet-induced dysbiosis is a possible approach in fighting the obesity epidemic [108]. Several researchers speak of the promising potential of gut microbiota-modifying therapies, such as probiotics, prebiotics, synbiotics or fecal microbiota transplants and the need for more research into these topics [67].

We argue that, first of all, we need to fix the food. We need to provide a better and more updated answer to the question: What should we eat? The question appears to have a rather simple answer provided by our evolutionary history: Eat mostly whole foods. This approach corresponds with findings from a wide range of nutritional studies where whole foods are consistently associated with good health.

## 9. Future Directions

We argue that we need to study how whole foods differ from the ultra-processed foods that are characteristic of the Western diet and how these foods differentially affect our microbiome. The characterization of our food supply is still incomplete in terms of predicting health effects.

Furthermore, as outlined in this paper, an average Western diet contains several stressors that have pathological potential as single factors. The sequence order, time frame and synchronicity of different environmental stressors, a few of which are discussed here, that have the potential to disrupt homeostatic balance in the human body are seldom emphasized. Models that take the “multiple hits” scenario into account are thus strongly needed to elucidate the total effects of a Western diet and how single factors interact with an environment containing multiple stressors.

Future research must aim at better identifying which aspects of food processing may impose negative health effects. Also, the use of possibly hazardous food additives calls for stronger regulation, and detrimental effects on the microbiome must be ruled out prior to approval. Research into nutrition has had its emphasis on the intake of micro and macronutrients, but recent studies of the food matrix have indicated that the package that the nutrients are delivered in appears to be just as important as the intake levels. Dietary advice should aim to improve the overall quality of the diet, rather than primarily focusing on single nutrients, and dietary advice should also emphasize the importance of the extent of processing. Brazil has pioneered this work, and, to our knowledge, was the first country to include advice to limit the intake of fast-foods and ultra-processed foods in their official dietary advice [109].

To improve food production practices, we propose a new, governmentally-directed labeling system, where processed foods are labeled by the level of processing, the addition of additives and other substitutions for raw material as well as by the percentage of whole foods present in the finished product. Such a system could facilitate increased taxation on ultra-processed foods. Tax income could subsidize whole and minimally processed foods, to enable these foods to stand out as better alternatives—also in low income populations. To minimize resistance from food industry lobbyists, such a system should be initiated at a transnational level. The United Nations General Assembly has proclaimed 2016–2025 the UN Decade of Action on Nutrition, aimed at fighting all forms of malnutrition and call for accelerated, coordinated, global action on nutrition [110]. In this context, our suggested labeling system could be a game-changer, providing a strong incentive for increased use of whole foods at the expense of processed food ingredients in future food production, to enable future symbiotic co-existence with our inner microbial world.

## Figures and Tables

**Table 1:** Components of a Western diet and effects on the microbiota and/or host physiology. This is not an exhaustive list. Examples are chosen based on a crude assessment of relevance to issues discussed in this paper (dosages administered are below or correspond to ADI levels, or are meant to mimic estimated intake levels in humans).

Food Additives	Effect on Microbiota	Effect on Host Physiology	Organism/Treatment	Reference
CMC	Bacterial overgrowth	Intestinal inflammation	Mice (IL10^−/−^). 2% CMC, 3 weeks	[54]
CMC, P-80	Microbiota encroachment, altered species composition, increased pro-inflammatory potential	Colitis, metabolic syndrome	Mice (IL10^−/−^, TLR5^−/−^). 1% CMC/P-80, 12 weeks Mice (WT). 0.1–1% CMC/P-80, 12 weeks	[29]
CMC, P-80	Increased pro-inflammatory potential		Human colon model. 1% CMC/P-80, duration: n/a	[33]
P-80	Microbiota encroachment, altered species composition, increased pro-inflammatory potential	Intestinal inflammation, obesity, liver dysfunction	Mice (WT). 1% P-80 per kg. bw, 4 weeks	[55]
GML	Gut microbiota dysbiosis	Metabolic syndrome, systemic low-grade inflammation	Mice (WT). 150 mg·kg^−1^ GML, 8 weeks	[56]
Titanium dioxide		Decrease in absorptive microvilli, decreased nutrient uptake	Human colon cells. 2.3 × 10^9^ (high), 2.3 × 10^7^ (medium), 2.3 × 10^5^ (low) particles/mL	[63]
Sucralose	Increased expression of bacterial pro-inflammatory mediators	Elevated pro-inflammatory gene expression in the liver	Mice (WT). 0.1 mg/mL sucralose, 6 months	[62]
NAS	Compositional and functional alterations of microbiota associated with obesity	Glucose intolerance	Mice (WT). 0.1 mg/mL^−1^ saccharin, 5 weeks	[28]
Saccharin	Increased pro-inflammatory potential	Liver inflammation	Mice (WT). 0.3 mg/mL saccharin, 6 months	[61]
Aspartame	Compositional alterations of microbiota	Glucose intolerance	Rats (WT). 5–7 mg/kg/d, 10 weeks	[59]
Acesulfame K	Compositional and functional alterations of microbiota associated with obesity	Weight gain (male)	Mice (CD-1). 37.5 mg/kg/d, 4 weeks	[60]
Silver nanoparticles	Gut microbial alterations associated with obesity and inflammatory diseases		Mice (WT). 46, 460 or 4600 ppb Ag NP, 28 days	[64]

Acceptable daily intake (ADI), Polysorbate 80 (P-80), Carboxymethylcellulose (CMC), Non-caloric artificial sweeteners (NAS), Glycerol Monolaureate (GML), Interleukin (IL), Toll like receptor (TLR), Wild type (WT), Not available (n/a), Body weight (bw), Cluster of differentiation 1 (CD1), Part per billion (ppb), Silver (Ag), Nanoparticles (NP).
